# Exploring the impact of suicide care experiences and post-intervention supports sought among community pharmacists: a cross-sectional survey

**DOI:** 10.1007/s11096-022-01398-4

**Published:** 2022-04-20

**Authors:** Sarira El-Den, Huai-Jin Choong, Rebekah J. Moles, Andrea Murphy, David Gardner, Alan Rosen, Claire L. O’Reilly

**Affiliations:** 1 The University of Sydney School of Pharmacy, Faculty of Medicine and Health, The University of Sydney, 2006 Sydney, New South Wales Australia; 2 College of Pharmacy, Faculty of Health, Dalhousie University, Halifax, Nova Scotia Canada; 3 Department of Psychiatry, Faculty of Medicine, Dalhousie University, Halifax, Nova Scotia Canada; 4 Brain & Mind Centre, The University of Sydney, Sydney, New South Wales Australia; 5 Illawarra Institute for Mental Health, University of Wollongong, Wollongong, New South Wales Australia

**Keywords:** Health Education, Mental Health, Pharmacists, Suicide

## Abstract

**Background:**

There is a need to appropriately train, support and remunerate pharmacists for their expanding roles in mental healthcare. Pharmacists often care for people experiencing mental health crises, including suicidal thoughts and behaviours, but little is known about pharmacists’ suicide care experiences.

**Aim:**

This cross-sectional study aimed to explore the impact of professional experiences with people at risk of suicide and support accessed, among community pharmacists.

**Method:**

A survey exploring pharmacists’ experiences with people at risk of suicide and post-intervention support-seeking was disseminated through Australian and Canadian professional associations, conferences and social media (June 2016-May 2017). Quantitative data were analysed using Chi-squared, Fisher’s exact and independent t-tests, where appropriate. Qualitative data exploring the impact of these experiences were thematically analysed, and reasons for not seeking help post-intervention were identified.

**Results:**

Among 378 respondents, 84% had encountered patients at risk of suicide and 28% had lost patients to suicide. Some were negatively affected personally and/or professionally (11%), of which 88% did not seek professional support, mainly due to uncertainty about available services. Pharmacists were significantly more negatively affected if they had a personal mental health diagnosis (p = 0.017) and previous suicide care experiences (p = 0.001). Qualitative themes included: expanding knowledge and skills, role limitation and emotional impact and response.

**Conclusion:**

A large proportion of pharmacists have interacted with suicidal patients and are impacted by these experiences, yet few seek help due to lack of awareness and access. There is a need to recognize pharmacists’ roles in suicide care, and develop pharmacist-specific post-intervention support.

## Impact statements

- Pharmacists’ experiences in caring for people at risk of suicide can result in negative personal and professional impacts.
- Pharmacists infrequently access support after caring for people at risk of suicide.
- Pharmacists cite lack of awareness of and access to available resources as some of the main barriers to accessing support after experiences in suicide care.

## Introduction

An estimated 800,000 people die by suicide annually [1], with a further 48–500 million exposed to suicide bereavement [2]. Suicide can have a profound impact on families and communities [3–5]. Those affected may experience grief, denial, blame, and question the reasons for suicide [4, 6]. Stigma surrounding suicide and suicide loss may lead people to isolate, or be shunned [7]. Symptoms of post-traumatic stress or other mental illnesses may develop, and those experiencing suicide loss are at higher risk of suicide [2, 4, 8, 9]. Suicide loss impacts a broad spectrum of healthcare professionals who have to cope with their own emotions of suicide loss and support the bereaved [10–12].

Up to 50–68% of those working in specialty mental healthcare have lost patients to suicide [13–15]. However, non-specialist mental health services may also be accessed prior to suicide deaths. Nearly 80% of people see a general practitioner within three months [16] and approximately 40% visit emergency services in the year prior to dying by suicide [17]. Severe distress was reported by 38% of psychologists, psychiatrists, and social workers following a patient suicide [18]. Distress among healthcare professionals can be amplified when grieving is limited by concerns over patient confidentiality, or legal advice against discussing the suicide [6, 19]. Fears of blame, litigation, and disrupted relationships with colleagues, as well as loss of confidence in clinical work have been reported [19, 20]. Previous research has explored the support available to healthcare professionals when a patient in their care dies, due to a broad range of causes [21–23]; however, there is limited research exploring the impact of providing suicide care on primary healthcare professionals.

Pharmacists are accessible primary care providers who interact with patients at risk of suicide [12, 24–30]. Pharmacists may have roles in suicide prevention such as through means restriction, given their roles as gatekeepers of medicines [12, 24–30]. Although the emerging literature demonstrates pharmacists interact with patients at risk of, and who die by suicide, only recently have concerns regarding support for pharmacists post-intervention been raised [12, 26]. For example, among pharmacists working in North Carolina 22.4% and 21.6% knew a patient who died by suicide or requested a lethal medication dose, respectively; however, 24.9% felt moderately/extremely uncomfortable talking with at-risk patients [30]. Nonetheless, to our knowledge, there is no research exploring pharmacists’ personal reactions and experiences post-intervention, specifically.

### Aim

This study aimed to explore pharmacists’ experiences of providing suicide care, focusing on the impact of these experiences and support sought by pharmacists.

### Ethics approval

 The study was approved by the Research Ethics Board at Dalhousie University (#2016–3832) and the Human Research Ethics Committee at The University of Sydney (#2016/464). Participants indicated consent by submitting their survey responses.

## Method

### Survey instrument

The online survey consisted of four sections, including demographics, and adaptations of the Attitude Towards Suicide Scale [31] and the Stigma of Suicide Scale Short Form [32]. Analyses regarding pharmacists’ stigma of suicide [33], and experiences with people at risk of suicide [34] have been published. This study focuses on data obtained through responses to the fourth section of the survey which was developed by content experts, including four pharmacists with expertise in mental health research of which one is a Mental Health First Aid (MHFA) Master Instructor. It was then reviewed by three psychiatrists, a nurse, two mental health consumers and a primary care physician to ensure it appropriately explored pharmacists’ general and most prominent experiences with people at risk of suicide, perceived barriers to providing suicide care, impact of experiences and support sought post-intervention. Analyses of most prominent experiences [12] and perceived barriers [34] have been published previously. This study explores the impact of suicide care experiences and post-intervention supports sought by pharmacists.

The survey was tested for face validity with five pharmacists, in Canada, and feedback guided amendments to improve language and clarity. The survey was self-administered online through Dalhousie University’s Opinio site (https://surveys.dal.ca/) from June 2016 to May 2017.

### Participants

Current and former community pharmacists in Australia and Canada were invited to complete the survey via an electronic link. Respondents were recruited via emails to Australian and Canadian professional associations, flyers distributed at conferences, word of mouth and social media. The sample for this manuscript includes participant pharmacists who completed section four of the online survey. Participating pharmacists were invited to enter a draw for a $150 supermarket gift card in their country.

### Data analyses

Quantitative data were imported to SPSS 24 [35]. Demographic variables (e.g. age, self-reported gender, years of work experience in community pharmacy, mental health crisis training, and personal mental illness diagnosis, or experience with a close contact living with mental illness and/or who had attempted or died by suicide) were chosen for their potential to influence behaviours and experiences [33] and the fact that crisis training, such as MHFA training, often incorporates content surrounding the need for MHFAiders to care for themselves [36]. Hence, prior training may impact experiences and behaviours following the provision of suicide care. Pharmacists who completed mental health crisis training were asked to indicate the program, which was coded as “MHFA” or “Other”. Participants who completed multiple programs were coded as “MHFA” if at least one was MHFA or coded as “Other” if none were MHFA. From section four, community pharmacists’ professional experiences in suicide care, the impact of these experiences, and personal help-seeking behaviors were included. Demographic characteristics of Canadian and Australian pharmacists were compared using chi-squared tests, Fisher’s exact tests and independent t-tests as appropriate.

Those encouraged to advance their knowledge and skills to support people experiencing mental illness(es) and/or mental health crises were categorized as “upskill in mental healthcare”. The time since the latest training completed was used to calculate the average duration since pharmacists completed training. Those negatively affected at a personal and/or professional level were categorized as “negative effects”, and lastly, a group not at all affected by experiences in suicide care.

Chi-squared tests were used (p < 0.05) to compare between pharmacists who were encouraged to upskill in mental healthcare, who experienced negative personal and/or professional effects, or were not at all affected. Fisher’s exact tests were used if any assumptions of the chi-squared test were violated [37].

Open-ended responses relating to the impact of suicide care and the reasons for not accessing professional support, personally by pharmacists, were qualitatively analysed. Open-ended responses were initially inductively coded by one author (HJC), before being categorized into themes in agreement with three other authors (SE, COR and RM). If an open-ended question gathered less than 10 responses, thematic analysis was not conducted.

## Results

### Quantitative survey results

#### Participant characteristics and previous training

There were 378 (out of 399) complete responses to section four. Analysis of demographic variables is presented in Table 1.

**Table 1:** Demographic information of community pharmacists and comparisons between Canada and Australia*

			Overall		Canada		Australia		p value^+^
	Sample size		378		223		155		
Means ± SD									
	Age		38.7	± 12.6	42.4	± 12.0	33.4	± 11.6	**< 0.0001**
	Years of experience in community pharmacy		14.2	± 12.3	17.1	± 12.4	9.9	± 10.9	**< 0.0001**
	Hours worked per week in community pharmacy		33.2	± 12.8	32.2	± 12.6	34.6	± 13.0	0.075
Frequencies									
	Sex								
		Male	110	(29%)	62	(28%)	48	(31%)	0.379
		Female	267	(71%)	161	(72%)	106	(68%)	
		Other	1	(0%)	0	(0%)	1	(1%)	
	Currently practicing as a community pharmacist		326	(86%)	184	(83%)	142	(92%)	**0.018**
	Geographic location								
		Remote	6	(2%)	2	(1%)	4	(3%)	**0.024**
		Rural	96	(25%)	67	(30%)	29	(19%)	
		Urban	276	(73%)	154	(69%)	122	(79%)	
	Position in the pharmacy								
		Pharmacist Employee	222	(59%)	141	(63%)	81	(52%)	**0.024**
		Pharmacist Manager	89	(24%)	52	(23%)	37	(24%)	
		Pharmacist Owner	67	(18%)	30	(13%)	37	(24%)	
	Ever diagnosed with a mental illness		110	(29%)	71	(32%)	39	(25%)	0.152
	Close friend or relative lives with a mental illness		268	(71%)	167	(75%)	101	(65%)	**0.021**
	Close friend or relative has attempted suicide or died from suicide		145	(38%)	87	(39%)	58	(37%)	0.705

*Canadian and Australian pharmacists were compared using Pearson chi-squared tests, Fisher’s exact tests and independent t-tests, as appropriate

^+^Significant associations are indicated by p-values in bold (< 0.05)

Pharmacists who had completed previous training in mental health crisis management had most commonly (41%) attended MHFA training (Table 2). Seven respondents had completed multiple training programs. Australian pharmacists were more likely to have mental health crisis training (p < 0.0001), to have completed MHFA (p < 0.0001), and to have guidance provided by their community pharmacy workplace to respond to mental health crises (p < 0.001) (Table 2).

**Table 2:** Mental health crisis training among community pharmacists and comparisons between Canada and Australia*

		Overall		Canada		Australia		p value^+^
	Sample size	378		223		155		
Previous training in mental health crisis management (n = 378)								
	Yes	71	(19%)	26	(12%)	45	(29%)	**< 0.0001**
	No	307	(81%)	197	(88%)	110	(71%)	
Name of training (n = 71)								
	Mental Health First Aid	29	(41%)	2	(8%)	27	(60%)	**< 0.0001**
	Other	42	(59%)	24	(92%)	18	(40%)	
Years since completing training (n = 70, mean ± SD)		2.64	± 2.31	3.42	± 2.88	2.19	± 1.78	0.057
How well did the training prepare you for supporting patients at risk of suicide? (n = 71)								
	Not at all	1	(1%)	1	(4%)	0	(0%)	0.721
	Somewhat	43	(61%)	16	(62%)	27	(60%)	
	Very well	21	(30%)	7	(27%)	14	(31%)	
	Unable to determine	6	(8%)	2	(8%)	4	(9%)	
How important is training in mental health crisis management for community pharmacists? (n = 378)								
	Not important	0	(0%)	0	(0%)	0	(0%)	0.461
	Somewhat important	104	(28%)	65	(29%)	39	(25%)	
	Very important	274	(72%)	158	(71%)	116	(75%)	
Community pharmacy provides guidance in responding to mental health crises (n = 378)								
	Yes	22	(6%)	4	(2%)	18	(12%)	**< 0.001**
	No	306	(81%)	189	(85%)	117	(75%)	
	Not sure	50	(13%)	30	(13%)	20	(13%)	

*Canadian and Australian pharmacists were compared using Pearson chi-squared tests and independent t-tests as appropriate

^+^Significant associations are indicated by p-values in bold (< 0.05)

#### Pharmacists’ experiences in suicide care

The majority (84%) of pharmacist respondents directly interacted with people at risk of suicide at least once; 38% provided care for patients who recently attempted suicide, and 28% had lost patients to suicide (Table 3).

**Table 3:** Community pharmacists’ experiences in suicide care^a^

Previously interacted with a person at risk of suicide							
0 times		61	(16%)				
1 or more times		317	(84%)				
		Yes		No		Not Sure	
Knew pharmacist colleagues with experience in suicide care (n = 317)		168	(53%)	60	(19%)	89	(28%)
Discussed with pharmacist colleagues about experiences in suicide care (n = 317)		166	(52%)	137	(43%)	14	(4%)
Cared for patient(s) with recent suicide attempt		142	(38%)	190	(50%)	46	(12%)
Directly acknowledged/discussed the issue of suicide with patients (n = 142)^b^		77	(54%)	57	(40%)	8	(6%)
Patient(s) they cared for died from suicide		104	(28%)	162	(43%)	112	(30%)
Years since most recent patient suicide death (n = 99, mean ± SD)		4.88	± 0.0561				

^a^ Means, SD and percentages were calculated out of 378 respondents unless sample sizes were specified otherwise

^b^ Pharmacists answered this question only if they have cared for patients with a recent suicide attempt

Table 4 describes how pharmacists were impacted by their experiences in suicide care. The majority were encouraged to upskill in providing care for people experiencing mental illness (74%) or mental health crises (62%). A small proportion were negatively affected personally (10%) and/or professionally (3%).

**Table 4:** Effects of experiences in suicide care on community pharmacists^*^

Effects of experiences with patients at risk of suicide			Yes		No	
Encouraged to advance knowledge and abilities in caring for and supporting…						
	(a) … people with mental illness and/or addictions		281	(74%)	97	(26%)
	(b) … people in a mental health crisis		233	(62%)	145	(38%)
	(c) Negative effect professionally		11	(3%)	367	(97%)
	(d) Negative effect personally		39	(10%)	339	(90%)
	Any of the four effects above(a-d)		347	(92%)	31	(8%)
	(e) Other (response in free-text)		57	(15%)	321	(85%)
Have you accessed professional support or care (n = 42)^**^			5	(12%)	37	(88%)
If no, why not? (n = 37)*						
	None recommended		7	(19%)		
	Not interested		12	(32%)		
	Lack of access		8	(22%)		
	Unsure where to get help		19	(51%)		
	Concerned about others knowing [they] sought help		1	(3%)		
	Other (open-ended response)		9	(24%)		

* Means, SD and percentages were calculated out of 378 respondents unless sample sizes were specified otherwise

**Pharmacists answered this question only if they reported negative personal and/or professional effects ((c) and/or (d)) following experiences in suicide care

#### Factors influencing impacts of experiences in suicide care

Pharmacists were more likely to seek to upskill in mental healthcare if they had previous training, previously interacted with patients at risk of suicide, were ever personally diagnosed with a mental illness or had a close contact who had attempted or died by suicide (all p < 0.05). No significant associations were found between how likely pharmacists reported negative effects and any demographic variables (Table 5).

**Table 5:** Factors influencing the likelihood of pharmacists to seek further training, or be negatively affected following experiences in suicide care

Characteristic		Encouraged to upskill in mental health care							Negative professional and/or personal effect					
		Yes (n = 331)		No (n = 47)		Chi square	P value		Yes (n = 42)		No (n = 336)		Chi square	P value
Previous mental health training														
	Yes	69	(97%)	2	(3%)	6.378	**0.012**		7	(10%)	64	(90%)	0.027	0.871
	No	262	(85%)	45	(15%)				35	(11%)	272	(89%)		
Previous interactions with people at risk of suicide														
	1 or more times	284	(90%)	33	(10%)	6.282	**0.012**		39	(12%)	278	(88%)	2.126	0.145
	0 times	47	(77%)	14	(23%)				3	(5%)	58	(95%)		
Ever diagnosed with a mental illness^a^														
	Yes	105	(95%)	5	(5%)	7.758	**0.005**		14	(13%)	96	(87%)	0.160	0.689
	No	222	(84%)	41	(16%)				28	(11%)	235	(89%)		
Close friend or relative with mental illness^a^														
	Yes	129	(89%)	16	(11%)	0.199	0.655		16	(11%)	129	(89%)	0.000	1.000
	No	198	(87%)	30	(13%)				26	(11%)	202	(89%)		
Close friend or relative attempted suicide or died by suicide^a^														
	Yes	266	(90%)	31	(10%)	4.018	**0.045**		34	(11%)	263	(89%)	0.001	0.981
	No	61	(80%)	15	(20%)				8	(11%)	68	(89%)		

Note: Data were presented as numbers and row percentages. N = 378 unless otherwise specified. Significant associations are indicated by p-values in bold (< 0.05)

^a^ n = 373 due to missing data

Pharmacists were significantly more likely to report negative effects if they had been personally diagnosed with a mental illness (p = 0.001), and if they previously interacted with patients at risk of suicide (p = 0.017) (Table 6).

**Table 6:** Factors influencing the impact of suicide care experiences among pharmacists

Characteristic		Negative professional and/or personal effects		Not at all affected		Chi square	p value
Previous mental health training							
	Yes	7	(88%)	1	(13%)	(-)^a^	0.127
	No	35	(54%)	30	(46%)		
Previous interactions with people at risk of suicide							
	1 or more times	39	(68%)	18	(32%)	10.665	**0.001**
	0 times	3	(19%)	13	(81%)		
Ever diagnosed with a mental illness^b^							
	Yes	14	(88%)	2	(13%)	5.740	**0.017**
	No	28	(50%)	28	(50%)		
Close friend or relative with mental illness^b^							
	Yes	16	(64%)	9	(36%)	0.212	0.645
	No	26	(55%)	21	(45%)		
Close friend or relative attempted suicide or died by suicide^b^							
	Yes	34	(63%)	20	(37%)	1.219	0.270
	No	8	(44%)	10	(56%)		

Note: n = 73. Significant associations are indicated by p-values in bold (< 0.05)

^a^ Fisher’s Exact test was used as an assumption of chi-squared test was violated (> 20% cells have expected count < 5 [40])

^b^ n = 72 due to missing data

#### Pharmacists’ help-seeking behaviours

Of those negatively affected, only 12% of pharmacists accessed professional support or care for themselves (Table 4). The most common reason pharmacists did not seek professional help was being unsure where to get help, followed by a lack of interest, lack of access, and having no sources of support recommended for pharmacists.

### Qualitative data synthesis

#### Impact of experiences in suicide care

Fifty-seven pharmacists further elaborated how their experiences in suicide care had affected them, personally and professionally, including their experiences in accessing further training and support. Qualitative analysis generated three themes: expanding knowledge and skills, role limitation, and emotional impact and response.

#### Expanding knowledge and skills

Community pharmacists felt that they often lacked the knowledge to appropriately care for people at risk of suicide:

*“Made me feel like a helpless bystander almost, only able to offer moral support. Better to say as little as possible, rather than the wrong thing.”* ID 571.

Hence, some expressed a need to advance their mental health knowledge and skills:

*“I realized how unequipped I was to deal with these situations and [it] has helped me want to pursue caring more for these patients and learning more about mental health.”* ID 670.

Despite pharmacists’ willingness to upskill in suicide care provision, barriers such, as lack of training options, were also identified:

*“I was encouraged but was not able to follow up with advancing my knowledge because there was no ‘push’ to learn (i.e. no readily available material to learn from).”* ID 719.

#### Role limitation

Some pharmacists felt their role in mental healthcare is limited due to time and system barriers:

*“I think that our role is not one to treat, due to time constraints.”* ID 158.

Other barriers to pharmacists’ roles included feeling that suicide care was outside of scope or “*beyond professional control*” (ID 555):

*“[T]he desire to help people with mental health … is difficult because the system see[s] you only as drug provider.”* ID 59.

#### Emotional impact and response

Pharmacists reported being emotionally impacted and experiencing a range of emotions and responses (e.g., anxiety, anger, trust, feeling sorry for patients, helplessness, relief, responsibility for advocacy) post-intervention, which impacted their practice, and shaped the way they sought personal support:

*“I had mild temporary anxiety because I was afraid the outcome would be bad, not enough for me to require help, but then when the patients got better, then I felt better.”* ID 113.

Some pharmacists were frustrated by their experiences:

*“… At first I was angry then just felt sorry for him* [the patient that died from suicide].*”* ID 337.

While others reflected on the need to develop improved referral and follow-up pathways:

*“There has to be a better way of follow-up to let these people know someone is looking after them and cares.”* ID 234.

Pharmacists also reflected on the reasons why people die by suicide, barriers to providing suicide care and what further actions could have prevented suicide deaths, despite patients appearing to have received optimal mental healthcare:

*“…a prescription is filled appropriately…and you’re left wondering if more could have been done…”* ID 391.

Furthermore, changes in clinical practice were reported whereby pharmacists expressed exercising more caution when assessing suicide risk in subsequent patients:

*“When dispensing antidepressants to a new pt.* [patient], *I worry about their degree of suicidality.”* ID 549.

#### Reflections on personal help-seeking

Ten pharmacists provided open-ended reflections relating to personal help-seeking, highlighting several barriers and enablers to personal help-seeking, including self-stigma, professional obligations and support from colleagues. Given the low number of responses, thematic analysis was not conducted; however, responses were reviewed and reported, where relevant. Pharmacists reported experiencing negative emotions reflecting self-stigma surrounding help-seeking:

*“Disappointment in myself for needing help and not being able to just deal with it myself*.” ID 335.

Pharmacists also reported prioritizing professional obligations and duty of care over personal health and wellbeing:

*“Staying focused on caring for the patients is the biggest obligation and I fear that access[ing] help for myself would put that obligation and duty in jeopardy*.” ID 59.

Some reported seeking informal help from colleagues:

*“The staff at the pharmacy got together to talk about it afterwards, which made me feel better*.” ID 543.

While others did not feel a need to seek support:

*“I just put it out of my mind and forgot about it*.” ID 508.

However, it is important to consider that help-seeking may have been influenced by patient outcomes:

*“Don’t feel I need help at this point. Knowing that the patient is doing better is great satisfaction*.” ID 577.

## Discussion

### Statement of key findings

This study explored the suicide care experiences of community pharmacists, with a focus on support sought post-intervention, demonstrating that these experiences impact Australian and Canadian pharmacists both personally and professionally. Pharmacists were encouraged to upskill in mental healthcare, yet few accessed personal professional support post-intervention, due to lack of awareness of available support and resources. This study highlights pharmacists’ post-intervention and postvention support needs and may guide the development of pharmacist-specific resources.

### Interpretation

Twenty-eight per cent of participating pharmacists reported losing a patient to suicide. Similarly, 22.4% of pharmacy staff in the US have had a patient die by suicide [30]. This is not unexpected, given that the majority of people who die by suicide have mental illness [38, 39], and pharmacists have diverse roles in psychotropic medicines supply [40], quality use of psychotropic medicines [41] and screening for mental illnesses [42], thereby caring for people living with mental illnesses regularly. Experiences of suicide care can have personal and professional impacts on healthcare professionals, as seen in studies involving trainee and consultant psychiatrists [13, 43]. In this study, negative effects were more likely to be reported among pharmacists who had previously interacted with patients at risk of suicide, and were personally diagnosed with a mental illness (Tables 5 and 6). Pharmacist participants reflected on what preventative actions they could have taken. Similarly, research among trainee psychiatrists found that continued thoughts about how the suicide could have been prevented were the most commonly reported adverse personal impacts following a patient suicide [43]. Nonetheless, healthcare curricula often lack sufficient education about suicide prevention, intervention and care [44]. There is a need to develop guidelines and resources for curricula integration, so that healthcare professionals can confidently care for people at risk of suicide, and access support post-intervention [44].

Over half of respondents reported discussing their experiences in suicide care with colleagues (Table 3), and some described seeking peer support by debriefing with colleagues. Similarly, among consultant psychiatrists, 85% described other psychiatrists and 93% described team members as helpful/very helpful sources of help after patient suicide [13] and 95% of trainee psychiatrists report discussing with colleagues [43]. Furthermore, psychiatrists and physicians have reported obtaining informal support from colleagues, family and friends, as a main strategy when coping with patient suicides [45, 46]. Among pharmacists (n = 42) who responded to the question enquiring about professional support sought, all had experienced negative effects post-intervention; however, 37 did not access any support (Table 4), with 51% unsure where to get help and 22% lacking access. While there are a range of Australian and Canadian mental health services, there may be a demand for and lack of awareness of pharmacist-specific support. Confidential telephone counselling for pharmacists is available in Australia [47] and the UK [48]. In Canada, there is no nationwide support service for pharmacists. However, provincial programs may exist, such as the Ontario Pharmacy Health Program [49] which provides local mental health support for pharmacists.

Participating pharmacists were significantly more likely to upskill in mental healthcare, after an experience in suicide care, if they had previous mental health crisis training, previously interacted with suicidal patients, or a personal experience of mental illness. However, even when these factors were absent, 77–87% still sought to upskill in mental healthcare. Suicide education is often lacking in healthcare curricula [50, 51], and primary healthcare professionals require further training [52, 53]. There are various suicide prevention training programs available for pharmacists; however, very few are compulsory [54]. There is a need for a minimum standard of mental health crisis education and training, to ensure pharmacists can confidently and comfortably care for patients experiencing mental health crises, including suicide. Training can be integrated in healthcare curricula [55–57], or enforced as a post-graduation requirement [54, 58]. Suicide training programs, delivered through continuing professional education, for example, can be effective for healthcare professionals [51]. Huh et al. [59] demonstrated that late-life suicide risk assessment training for healthcare professionals was successful in improving confidence, knowledge, case note quality, and recognition and awareness of suicide risk, as well as changing clinical practice when assessing and managing suicide risk [59]. There is a growing global push to make suicide assessment and prevention training mandatory for primary healthcare professionals, with pharmacists in Washington state now required to complete mandatory training [58].

Participating Canadian (12%) and Australian pharmacists (29%) had completed mental health crisis training, of which 8% and 60% had completed MHFA training, respectively. Similarly, among 7% of US pharmacists who had completed suicide prevention training, 37.1% had completed MHFA [30]. MHFA training teaches the provision of immediate support to people experiencing mental health problems or crises [36]. Significantly higher proportions of Australian pharmacists reported completing MHFA training, which may be expected as the program was established in Australia in 2000 [36]. MHFA improves mental health knowledge, attitudes and skills among diverse populations [60], including pharmacy students [61]. Adequate training may also be important for pharmacists’ own personal benefit as evidence suggests that healthcare professionals with less training and clinical practice experience are more likely to experience more severe reactions and distress in relation to patient suicides [6].

Pharmacists who know how to help someone who is suicidal are significantly more likely to provide a suicide assessment [62]. Given that MHFA is effective, and there is high uptake of MHFA training among pharmacists [30], there may be a need to integrate MHFA into healthcare curricula or render it a requirement for healthcare professionals on registration, similar to physical first aid training [57]. Community pharmacy staff have indicated their preference for gatekeeper training that includes role-play scenarios reflecting “realistic interactions” [63]; hence, assessments allowing for observation and participation, such as simulated patient role-plays with immediate debrief and feedback, may also be required [53, 56, 57] to improve self-efficacy by providing an opportunity to practice newly acquired skills and interact with consumers in safe learning environments.

Feelings of uncertainty around healthcare professionals’ roles in suicide care and prevention are common among a diverse range of healthcare professionals, including physicians [64]. Perhaps uncertainty regarding possible outcomes after consumers leave the pharmacy and variability as to whether pharmacists will be notified of suicide attempts and completed suicides are additional stresses which weigh heavily on the minds of involved and caring pharmacists. Participating pharmacists perceived “role limitation” to be a barrier to their expanding roles in suicide care. Internationally, pharmacists’ roles are expanding beyond that of medicines supply, to include public health services such as health promotion, as well as, disease prevention and management [65]. A criticism is often that the evidence base for these expanded roles is lacking [65]. This study helps build the evidence base to ensure pharmacists’ roles in mental healthcare are recognized, supported and remunerated. Up to 85% of pharmacists report that they have come into contact with people at risk of suicide and they often go beyond what is considered to be their traditional medicine-related roles, to intervene, refer and follow-up where necessary [12]. Policies and guidelines outlining pharmacists’ integral roles in mental healthcare are emerging in pharmacy-based literature [41, 66]; however, pharmacists’ contribution to mental healthcare is often missing from national mental health guidance and literature, both in Australia [67] and Canada [68].

### Further research

Finally, there are various barriers that may impede pharmacists’ roles in suicide care. Time constraints is a common barrier to assessing suicide risk [62] and to having discussions about suicide with patients [30]. Hence, it is not surprising that pharmacists in the current study perceived they have limited roles in mental healthcare, when the opposite is true. Community pharmacists need to be recognised and integrated within healthcare systems to support their increasing role in mental healthcare, including suicide prevention, at a national and international level. The public needs to be aware of these roles, and there needs to be appropriate remuneration for these services [65].

### Strengths and weaknesses

This is the first study exploring pharmacists’ post-intervention experiences, the impact on their personal and professional lives and supports sought by pharmacists. Although, the survey was developed by content experts and tested for face validity by pharmacists, further psychometric testing is warranted. Furthermore, the study focused on pharmacists’ roles in suicide care and the questions were not specific to pharmacists who had lost patients to suicide. Most participating pharmacists had not lost patients to suicide; hence, future research focusing specifically on postvention supports are needed.

Owing to the nature of online surveys and the anonymity of responses, it was not possible to calculate a response rate. Moreover, respondents may have been more likely to participate due to existing interests and/or experiences in mental healthcare. Hence, the potential for volunteer bias may overestimate how commonly pharmacists provide suicide care.

Finally, it is also important to note that the data presented in this manuscript was collected in 2016–2017. Recent studies from the US and UK have also demonstrated that a high proportion of pharmacy staff interact with people at risk of suicide and that pharmacy staff require further training to appropriate support this vulnerable population [30, 69, 70]. However, there is still a gap in the literature exploring the impact of suicide care experiences and post-intervention supports among pharmacists, in that no studies have focused on this topic. Hence, while there is a need to consider the age of the data, the lack of research in this area highlights the need for further studies exploring pharmacists’ experiences after assisting with mental health crises and the importance of this study, as it is the first to explore this topic specifically.

## Conclusion

Pharmacists are impacted personally and professionally by suicide care experiences and suicide loss, yet few seek professional help. Pharmacists acknowledge the need to advance their knowledge and skills to ensure they are able to appropriately and safely recognize, assess and care for patients at risk of suicide. These findings may inform future post-intervention and postvention research among pharmacists, as well as primary healthcare professionals generally.

## References

[1] World Health Organisation. Suicide data 2016. Available from: https://www.who.int/mental_health/prevention/suicide/suicideprevent/en/. Accessed 23 Sep 2019.

[2] Pitman A, Osborn D, King M (2014). Effects of suicide bereavement on mental health and suicide risk. Lancet Psychiatry.

[3] Cerel J, Aldrich RS (2011). The impact of suicide on children and adolescents. Grief after suicide: Understanding the consequences and caring for the survivors. Series in death, dying and bereavement.

[4] Jordan JR (2017). Postvention is prevention—The case for suicide postvention. Death Stud.

[5] Sandler EP. The Ripple Effect Of Suicide 2018. Available from: https://www.nami.org/Blogs/NAMI-Blog/September-2018/The-Ripple-Effect-of-Suicide. Accessed 07 Nov 2019.

[6] Al-Mateen CS, Jones K, Linker J, O’Keefe D, Cimolai V (2018). Clinician Response to a Child Who Completes Suicide. Child Adolesc Psychiatr Clin N Am.

[7] Sveen CA, Walby FA (2008). Suicide survivors’ mental health and grief reactions: a systematic review of controlled studies. Suicide Life-Threat Behav.

[8] Stroebe M, Stroebe W, Abakoumkin G (2005). The broken heart: suicidal ideation in bereavement. Am J Psychiatry.

[9] Jordan JR, McIntosh JL. Suicide bereavement: Why study survivors of suicide loss? Grief after suicide: Understanding the consequences and caring for the survivors. Routledge/Taylor & Francis Group; 2011. pp. 3–17.

[10] General Practice Mental Health Standards Collaboration (GPMHSC). Suicide prevention and first aid: A resource for GPs. East Melbourne, Vic; 2016.

[11] General Practice Mental Health Standards Collaboration (2016). After suicide: A resource for GPs.

[12] Murphy A, Ataya R, Himmelman D (2018). Community pharmacists’ experiences and people at risk of suicide in Canada and Australia: a thematic analysis. Soc Psychiatry Psychiatr Epidemiol.

[13] Alexander DA, Klein S, Gray NM (2000). Suicide by patients: questionnaire study of its effect on consultant psychiatrists. BMJ.

[14] Chemtob CM, Hamada RS, Bauer G (1988). Patients’ suicides: frequency and impact on psychiatrists. Am J Psychiatry.

[15] Wurst FM, Mueller S, Petitjean S (2010). Patient suicide: a survey of therapists’ reactions. Suicide Life-Threat Behav.

[16] De Leo D, Draper BM, Snowdon J (2013). Contacts with health professionals before suicide: Missed opportunities for prevention?. Compr Psychiatr.

[17] Gairin I, House A, Owens D (2003). Attendance at the accident and emergency department in the year before suicide: Retrospective study. Br J Psychiatry.

[18] Hendin H, Haas AP, Maltsberger JT, Szanto K, Rabinowicz H (2004). Factors contributing to therapists’ distress after the suicide of a patient. Am J Psychiatry.

[19] Gutin N, McGann VL, Jordan JR. The Impact of Suicide on Professional Caregivers. Grief after Suicide: Understanding the Consequences and Caring for the Survivors. Taylor and Francis; 2011. pp. 93–111.

[20] Cotton P, Drake R, Whitaker A (1983). Dealing with suicide on a psychiatric inpatient unit. Hosp Community Psychiatry.

[21] Keene EA, Hutton N, Hall B (2010). Bereavement debriefing sessions: an intervention to support health care professionals in managing their grief after the death of a patient. Pediatr Nurs.

[22] Redinbaugh EM, Sullivan AM, Block SD (2003). Doctors’ emotional reactions to recent death of a patient: cross sectional study of hospital doctors. BMJ.

[23] Moores TS, Castle KL, Shaw KL (2007). ‘Memorable patient deaths’: reactions of hospital doctors and their need for support. Med Educ.

[24] Murphy AL, Hillier K, Ataya R (2017). A scoping review of community pharmacists and patients at risk of suicide. Can Pharmacists J / Revue des Pharmaciens du Can.

[25] Murphy AL, O’reilly C, Martin-Misener R (2018). Community pharmacists’ attitudes on suicide: A preliminary analysis with implications for medical assistance in dying. Can Pharmacists J / Revue des Pharmaciens du Can.

[26] Murphy AL, Gardner DM, Chen TF (2015). Community pharmacists and the assessment and management of suicide risk. Can Pharmacists J / Revue des Pharmaciens du Can.

[27] Kassir H, Eaton H, Ferguson M, et al. Role of the pharmacist in suicide prevention: primely positioned to intervene. J Pharm Pract Res. 2019;49(6):567–69.

[28] Finkelstein Y, Macdonald EM, Hollands S (2015). Risk of Suicide Following Deliberate Self-poisoning. JAMA psychiatry.

[29] Sinyor M, Howlett A, Cheung AH, Schaffer A (2012). Substances Used in Completed Suicide by Overdose in Toronto: An Observational Study of Coroner’s Data. Can J Psychiatry.

[30] Carpenter DM, Lavigne JE, Colmenares EW (2020). Community pharmacy staff interactions with patients who have risk factors or warning signs of suicide. Res Social Administrative Pharm.

[31] Renberg ES, Jacobsson L (2003). Development of a questionnaire on attitudes towards suicide (ATTS) and its application in a Swedish population. Suicide Life-Threat Behav.

[32] Batterham PJ, Calear AL, Christensen H (2013). The Stigma of Suicide Scale. Psychometric properties and correlates of the stigma of suicide. Crisis.

[33] Murphy AL, O’Reilly CL, Ataya R, et al. A survey of Canadian and Australian pharmacists’ stigma of suicide. SAGE Open Medicine. 2019;7. 10.1177/2050312118820344 PMC6350138 30728964

[34] Murphy AL, O’Reilly CL, Ataya R (2019). Survey of Australian and Canadian Community Pharmacists’ Experiences With Patients at Risk of Suicide. Psychiatric Serv.

[35] IBM Corp (2016). IBM SPSS Statistics for Windows, Version 24.0.

[36] Kitchener BA, Jorm AF, Kelly CM. Mental Health First Aid Manual, Fourth Edition. Parkville, Victoria: Mental Health First Aid Australia; 2017.

[37] Pallant JF (2011). Non-parametric statistics. SPSS survival manual: a step by step guide to data analysis using the SPSS program.

[38] Hirschfeld RMA, Russell JM (1997). Assessment and Treatment of Suicidal Patients. N Engl J Med.

[39] Cavanagh JT, Carson AJ, Sharpe M (2003). Psychological autopsy studies of suicide: a systematic review. Psychol Med.

[40] Australian Institute of Health and Welfare (2019). Mental Health Services in Australia. Mental health-related prescriptions.

[41] Pharmaceutical Society of Australia. Mental health care project: A framework for pharmacists as partners in mental health care. 2013 February 2013.

[42] O’Reilly CL, Wong E, Chen TF (2015). A feasibility study of community pharmacists performing depression screening services. Res social administrative pharmacy: RSAP.

[43] Dewar I, Eagles J, Klein S (2000). Psychiatric trainees’ experiences of, and reactions to, patient suicide. Psychiatr Bull.

[44] Henry J, Ramages M, Cheung G (2020). The development of patient suicide post-vention guidelines for psychiatry trainees and supervisors. Australasian Psychiatry.

[45] O’Dowd E, O’Connor P, Lydon S (2018). Stress, coping, and psychological resilience among physicians. BMC Health Serv Res.

[46] Hawgood J, Leo DD. Working with suicidal clients: Impacts on psychologists and the need for self-care. InPsych. 2015;37(1).

[47] Pharmacists Support Services. About Us 2019 [Available from: https://www.supportforpharmacists.org.au/index.php/about-us-2/about-pss]. Accessed 31 Oct 2019.

[48] Pharmacist Support. Pharmacist Support: Working for Pharmacists & their Families [Available from: https://pharmacistsupport.org/]. Accessed 31 Oct 2019.

[49] Lifemark Workplace Health & Wellness. Ontario Pharmacy Health Program 2019 [Available from: https://www.lifemarkworkhealth.ca/services/disability-management/ontario-pharmacy-health-program]. Accessed 23 Sep 2019.

[50] Boukouvalas E, El-Den S, Murphy AL, et al. Exploring health care professionals’ knowledge of, attitudes towards and confidence in caring for people at risk of suicide: a systematic review. Arch Suicide Res. 2020; 24(sup2), S1-S31. 10.1080/13811118.2019.1586608 30856366

[51] Schmitz WM Jr, Allen MH, Feldman BN, et al. Preventing Suicide through Improved Training in Suicide Risk Assessment and Care: An American Association of Suicidology Task Force Report Addressing Serious Gaps in U.S. Mental Health Training. Suicide Life Threat Behav. 2012;42(3):292–304. 10.1111/j.1943-278X.2012.00090.x 22494118

[52] Gaynes BN, West SL, Ford CA (2004). Screening for Suicide Risk in Adults: A Summary of the Evidence for the U.S. Preventive Services Task Force. Ann Intern Med.

[53] Sher L (2011). Teaching medical professionals about suicide prevention: what’s missing?. QJM: An International Journal of Medicine.

[54] Carpenter DM, Lavigne JE, Roberts CA (2018). A review of suicide prevention programs and training policies for pharmacists. J Am Pharmacists Association.

[55] Boukouvalas EA, El-Den S, Chen TF (2018). Confidence and attitudes of pharmacy students towards suicidal crises: patient simulation using people with a lived experience. Soc Psychiatry Psychiatr Epidemiol.

[56] El-Den S, Chen TF, Moles RJ (2018). Assessing Mental Health First Aid Skills Using Simulated Patients. Am J Pharm Educ.

[57] El-Den S, Moles R, Choong HJ (2020). Mental Health First Aid training and assessment among university students: A systematic review. J Am Pharm Assoc.

[58] Washington State Department of Health. Suicide Prevention Training for Health Professionals 2020 [Available from: https://www.doh.wa.gov/ForPublicHealthandHealthcareProviders/HealthcareProfessionsandFacilities/SuicidePrevention/TrainingRequirements]. Accessed 08 Dec 2020.

[59] Huh JT, Weaver CM, Martin JL (2012). Effects of a Late-Life Suicide Risk–Assessment Training on Multidisciplinary Healthcare Providers. J Am Geriatr Soc.

[60] Morgan AJ, Ross A, Reavley NJ (2018). Systematic review and meta-analysis of Mental Health First Aid training: Effects on knowledge, stigma, and helping behaviour. PLoS ONE.

[61] O’Reilly CL, Bell JS, Kelly PJ (2011). Impact of mental health first aid training on pharmacy students’ knowledge, attitudes and self-reported behaviour: a controlled trial. Aust N Z J Psychiatry.

[62] Gillette C, Mospan CM, Benfield M (2020). North Carolina community pharmacists’ attitudes about suicide and willingness to conduct suicidal ideation assessment: A cross-sectional survey study. Res Social Administrative Pharm.

[63] Carpenter DM, Roberts CA, Lavigne JE (2021). Gatekeeper training needs of community pharmacy staff. Suicide Life-Threat Behav.

[64] Boeke M, Griffin T, Reidenberg DJ (2011). The physician’s role in suicide prevention: lessons learned from a public awareness campaign. Minn Med.

[65] Mossialos E, Naci H, Courtin E (2013). Expanding the role of community pharmacists: Policymaking in the absence of policy-relevant evidence? Health policy (Amsterdam. Netherlands).

[66] International Pharmaceutical Federation (FIP) (2015). Focus on mental health: the contribution of the pharmacist.

[67] National Mental Health Strategy. The Fifth National Mental Health and Suicide Prevention Plan. 2017.

[68] Canadian Association for Suicide Prevention (2009). The CASP Blueprint for a Canadian National. Suicide Prev Strategy.

[69] Gorton HC, O’Reilly C, Berry HJ, Babar, Z-U-D (2021). Chapter 6 - Understanding pharmacy staff attitudes and experience relating to suicide. Pharmacy Practice Research Case.

[70] Gorton HC, Littlewood D, Lotfallah C (2019). Current and potential contributions of community pharmacy teams to self-harm and suicide prevention: A qualitative interview study. PLoS ONE.

